# Antibiotic Prophylaxis in Patients Undergoing Lung Transplant: Single-Center Cohort Study

**DOI:** 10.3389/ti.2024.13245

**Published:** 2024-08-16

**Authors:** Renato Pascale, Beatrice Tazza, Armando Amicucci, Elena Salvaterra, Giampiero Dolci, Filippo Antonacci, Massimo Baiocchi, Saverio Pastore, Simone Ambretti, Maddalena Peghin, Pierluigi Viale, Maddalena Giannella

**Affiliations:** ^1^ Department of Medical and Surgical Sciences, Alma Mater Studiorum University of Bologna, Bologna, Italy; ^2^ Infectious Diseases Unit, Scientific Institute for Research, Hospitalization and Healthcare Azienda Ospedaliero-Universitaria di Bologna, Bologna, Italy; ^3^ Division of Interventional Pulmonology Unit, Scientific Institute for Research, Hospitalization and Healthcare Azienda Ospedaliero-Universitaria di Bologna, Bologna, Italy; ^4^ Thoracic Surgery, Azienda Ospedaliero Universitaria di Ferrara, Ferrara, Italy; ^5^ Thoracic Surgery, Scientific Institute for Research, Hospitalization and Healthcare Azienda Ospedaliero-Universitaria di Bologna, Bologna, Italy; ^6^ Anaesthesiology and Intensive Care, Scientific Institute for Research, Hospitalization and Healthcare Azienda Ospedaliero-Universitaria Di Bologna, Bologna, Italy; ^7^ Microbiology Unit, Scientific Institute for Research, Hospitalization and Healthcare Azienda Ospedaliero-Universitaria di Bologna, Bologna, Italy; ^8^ Infectious and Tropical Diseases Unit, Department of Medicine and Surgery, University of Insubria-ASST-Sette Laghi, Varese, Italy

**Keywords:** lung transplant, antibiotic prophylaxis, bacterial infection, donor derived infections, idiopatic pulmonary fibrosis

## Abstract

Perioperative antibiotic prophylaxis (PAP) in lung transplant recipients (LuTRs) has high heterogeneity between centers. Our aim was to investigate retrospectively the approach to PAP in our center over a 20-year period (2002–2023), and its impact on early post-operative infections (EPOIs) after lung transplantation (LuT). Primary endpoint was diagnosis of EPOI, defined as any bacterial infection including donor-derived events diagnosed within 30 days from LuT. Main exposure variables were type of PAP (combination vs. monotherapy) and PAP duration. We enrolled 111 LuTRs. PAP consisted of single-agent or combination regimens in 26 (25.2%) and 85 (74.8%) LuTR. Median PAP duration was 10 days (IQR 6–13) days. Piperacillin/tazobactam was the most common agent used either as monotherapy (n = 21, 80.7%) or as combination with levofloxacin (n = 79, 92.9%). EPOIs were diagnosed in 30 (27%) patients. At multivariable analysis no advantages were found for combination regimens compared to single-agent PAP in preventing EPOI (OR: 1.57, 95% CI: 0.488–5.068, p:0.448). The impact of PAP duration on EPOIs development was investigated including duration of PAP ≤6 days as main exposure variables, without finding a significantly impact (OR:2.165, 95% CI: 0.596–7.863, p: 0.240). Our results suggest no advantages for combination regimens PAP in preventing EPOI in LuTR.

## Graphical Abstract

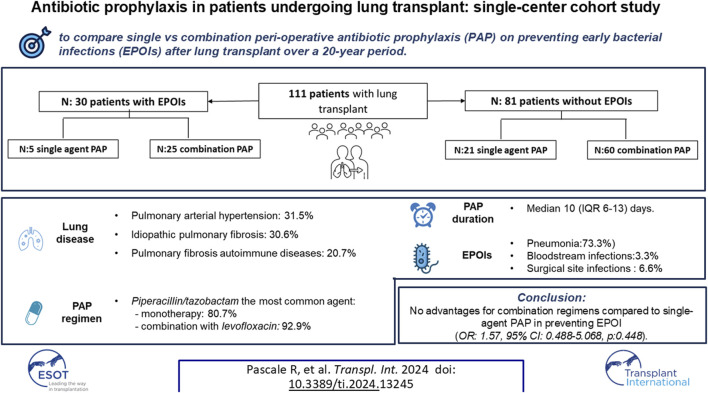

## Introduction

Bacterial infections are clinically relevant complications in lung transplant recipients (LuTRs) causing chronic lung allograft dysfunction and high rates of mortality, especially within the first year after transplant [1, 2]. Infectious episodes occurring in the first 30 days following lung transplantation (LuT) are due to microorganisms deriving from the donor lung or pre-existing recipient flora [3]. Indeed, even though native lungs are removed, colonization of the grafts from recipients colonizing strains often rapidly occurs [4–6]. While some experts advise against the use of the organs with microorganism isolation or detection, others support it, if it is combined with at least 24 h of antibiotic therapy according to susceptibility patterns of recovered microorganisms [7]. This last approach is supported by retrospective studies showing no difference in overall survival of recipients of infected/colonized lungs, compared to recipients of uninfected lungs [8], even in case of multi-drug resistant organisms (MDRO). All this considered, antibiotic prophylaxis is routinely administered in LuTRs [8, 9]. International guidelines recommendations are generic and predominantly based on cardiac procedures and no formal recommendations to guide antimicrobial selection in LuT surgery are currently available [10]. Therefore, antibiotic regimens for peri-operative antibiotic prophylaxis (PAP) are based on clinical judgment, bacterial infection and/or colonization present in the donor and/or recipient and knowledge of the local epidemiology, inducing high heterogeneity, both for drug choice and treatment duration, in clinical practice between centers [7].

We aim to carry-out a retrospective observational study to investigate different regimens of PAP used in our center over 20-year period, and their impact on preventing early bacterial infections and donor derived infection after LuT. The results obtained from our study will contribute to increasing knowledge about the prophylaxis regimens that can be adopted in hospitals with a similar clinical and microbiological epidemiology.

## Materials and Methods

### Study Design and Setting

Retrospective, monocentric observational cohort study including all adult patients who underwent LuT at IRCCS Azienda Ospedaliero-Universitaria di Bologna from 1st January 2002 to 31st August 2023. During the study period, LuTRs antibiotic prophylaxis regimens were established by Transplant Intensivists and Pneumologists who managed the patients in the immediate peri-transplant period, according to usual practice and internal guidelines.

All enrolled patients are followed from time of LuT to 30 days after (or until death, whichever occurred first). Data were retrospectively collected from medical charts and microbiology archives. Data were collected using a dedicated REDCap electronic case report form (eCRF) hosted by IRCCS Azienda Ospedaliero-Universitaria di Bologna [11]. The study was conducted according to declaration of Helsinki and Good Clinical Practice guidelines and approved by the local Ethics Committee (no 676/2023/Oss/AOUBo).

### Population

All adult (≥18 years) patients who underwent LuT receiving PAP during the study period were screened for inclusion. Patients were excluded in case of lack of clinical and/or laboratory data regarding type of early postoperative bacterial infections (EPOIs).

### Procedures

During the study period PAP regimen was composed by piperacillin-tazobactam administered with 9 g as loading dose followed by 4.5 g every 6 h in combination with levofloxacin 500 mg every 12 h [12–14]. This PAP regimen was chosen to provide two drugs with potential activity against *Pseudomonas* spp while awaiting donor/recipient culture results. PAP duration was continued until results of perioperative cultures. In case of positive recipient and/or donor cultures, PAP was tailored and extended for 10–14 days. In case of penicillin-drug allergy or intolerance, piperacillin/tazobactam was replaced by cefepime or meropenem. Levofloxacin was not administered in case of drug allergy, presence of a contraindication (history of epilepsy, connective disease, QT prolongation), or according with clinical judgement. When available, the pre-transplant recipient colonization status, at upper or lower respiratory tract, was used to target the antibiotic prophylaxis.

After transplantation, lower respiratory tract samples were taken when patients had clinical signs or symptoms of respiratory tract infection or rejection. During all the study period,in clinically stable patients, post-transplant bronchoscopy was performed after 48 h post-transplantation and subsequently every week for the first month. During each procedure, bronchoalveolar lavage and transbronchial biopsy were sent for microbiological cultures.

### Variables and Definitions

Primary endpoint was diagnosis of EPOIs, defined as any bacterial infection diagnosed according to US Centers for Disease Control and Prevention (CDC) criteria [15] within 30 days from LuT. Among EPOIs, donor derived events were included and defined as any bacterial infection caused by the same microorganism isolated from the donor and the recipient [16].

PAP was defined as the antibiotic regimen administered at time of LuT before susceptibility report of donor cultures were available. Changes in antibiotic treatment according with recipient/donor cultures were recorded as well as the overall duration of PAP. PAP was mainly classified as single-agent or combination regimen.

The other variables were age and sex, co-morbidities summarized by the Charlson Comorbidity score [17], information on LuT (transplant date, graft Number; graft function during first 24 h). Immunosuppressive drugs used as induction and maintenance regimen were recorded. Donor and recipient colonization were inferred from respiratory samples. Infection etiology was also classified according to the causative species into Gram positives and Gram negative rods. According to the definitions of CDC [18] all strains from donors/recipients were categorized as Oxacillin-resistant (OxaR) or Vancomicin Resistant (VR) for Gram positive and Carbapenem resistance (CR), third generation cephalosporin resistance (ESCR), β-lactam/β-lactamase inhibitor (BL/BLIR) and fluroquinolone resistance (FQR) for Gram negatives. For the latter, Magiorakos criteria (non-MDR, MDR, XDR or PDR) and the new definition of *“difficult to treat resistance”* (DTR) were also used [19, 20].

### Microbiology

Clinical samples collected during the study period were analysed following routine diagnostic workflow in the bacteriology laboratory, Unit of Microbiology. Since 2010, donor derived samples collected at the time of organ removal were referred to our center to be analyzed. Before that time, all donor cultures were performed at donor center and results subsequently provided at Transplant Unit. Results of any other microbiological samples previously analyzed at the donor center were provided to our center through the regional transplant coordination network.

### Statistical Analysis

For descriptive analysis, categorical variables were presented as frequencies and percentages, continuous variables were presented as mean ± standard deviation (SD) or median and interquartile range (IQR) according to normal or non-normal distribution. The clinical and demographic characteristics of the two subgroups of the study population were described and compared using bivariate tests such as t-test or Mann-Whitney test for continuous variables, chi-square or Fisher’s exact test for categorical variables.

A multivariable binary logistic regression analysis was performed to investigate independent predictors of EPOIs considering type of PAP regimen (single-agent vs. combination) as main exposure variable along with all the other variables showing a p < 0.05 at univariable analysis, including male gender, Charlson Comorbidity Index, Tacrolimus as maintenance regimen, idiopatic pulmonary fibrosis as leading cause for lung transplant and primary graft non function. A second model to assess the impact of PAP duration on EPOIs development was done in which patients on PAP at the time of infection diagnosis were excluded. For this analysis we used the cutoff of PAP duration ≤6 vs. >6 days considering that in most cases cultures results of donor and recipient samples were available and communicated within 6 days of transplantation. SPSS 21.0 was used for all analyses, with significance level set at α = 0.05.

## Results

**FIGURE 1:**
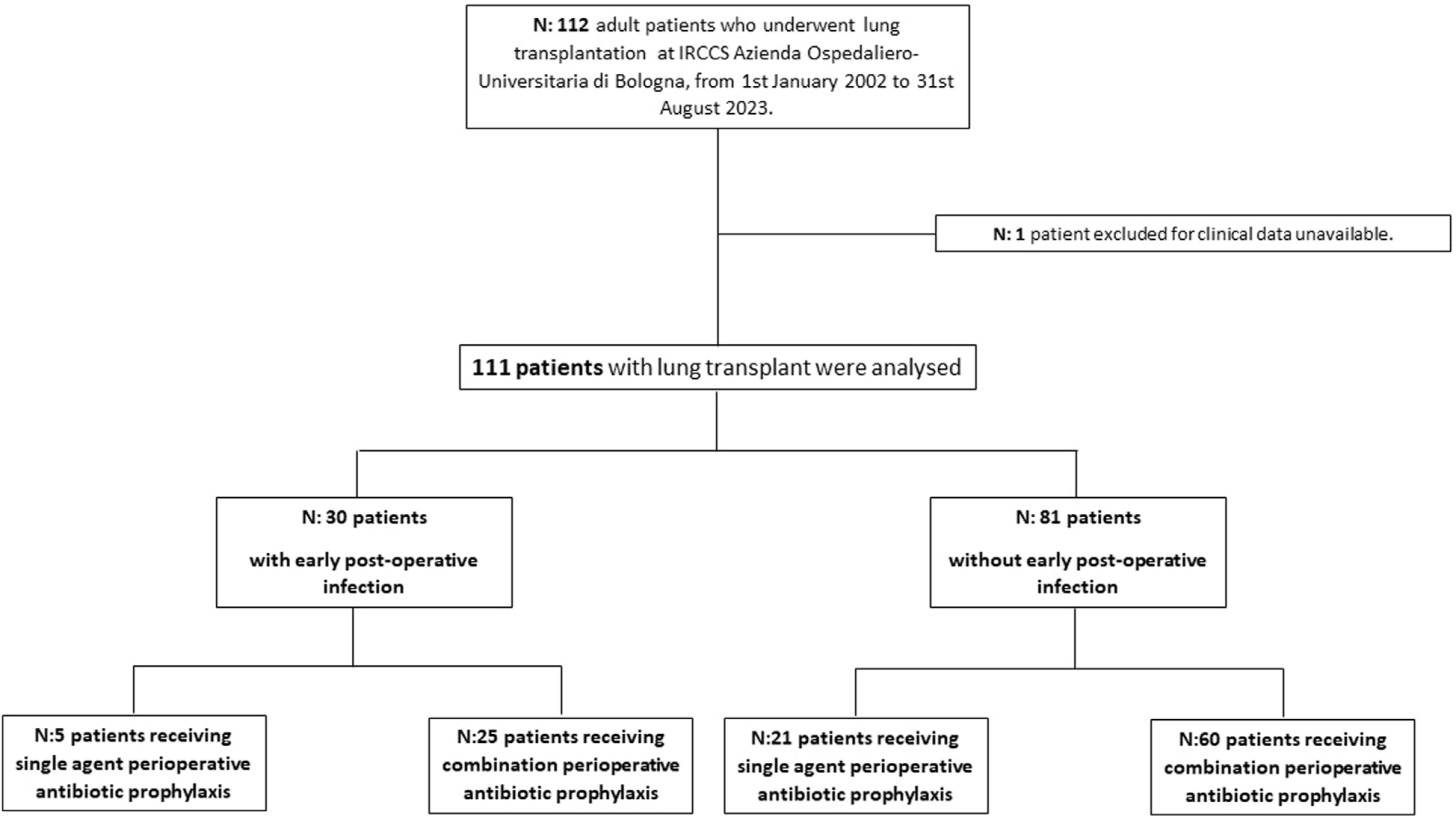
Study flow chart.

Overall, 112 patients receiving LuT were screened for inclusion, 1 patient was excluded for lack of data and 111 were analysed (Figure 1). The main characteristics of study population are shown in Table 1. Of them, 64 (57.5%) were male, with a median age of 50 years (IQR 39–59), median Charlson Index was 2 (IQR:1–4). The most frequent underlying lung disease leading to LuT was pulmonary arterial hypertension (35, 31.5%), followed by idiopathic pulmonary fibrosis (IPF) (34, 30.6%), pulmonary fibrosis associated with autoimmune diseases (23, 20.7%) and emphysema and chronic obstructive pulmonary disease (COPD) (14, 12.6%). Patients with cystic fibrosis were absent in our cohort. Primary graft non function was experienced by 15 (14%) patients.

**TABLE 1:** Characteristics of patients receiving lung transplant and comparison of patients with and without bacterial infection after lung-transplant.

	Cases with available data	Total of patient 111 (%)	Patients without bacterial infection 81 (%)	Patients with bacterial infection 30 (%)	p-value
Demographics					
Age (years), median (IQR)	111	50 (39–59)	48 (36–59)	54 (46–63)	0.029
Gender (male)	111	64 (57.5)	42 (51.9)	22 (73.3)	0.042
Underlying Lung Disease					
Idiopatic pulmonary fibrosis	111	34 (30.6)	20 (24.7)	14 (46.7)	0.026
Pulmonary fibrosis associated with autoimmune diseases	111	23 (20.7)	17 (21.0)	6 (20.0)	0.909
Emphysema/COPD	111	14 (12.6)	11 (13.6)	3 (10.0)	0.614
Pulmonary arterial hypertension	111	35 (31.5)	27 (33.3)	8 (26.7)	0.502
Chronic Thromboembolic Pulmonary Hypertension	111	1 (0.9)	1 (1.2)	0 (0.0)	0.541
Other	111	10 (9.0)	9 (11.1)	1 (3.3)	0.204
Underlying diseases					
Myocardial infarction	111	6 (5.4)	4 (4.9)	2 (6.7)	
Congestive heart failure	111	45 (40.5)	28 (34.6)	17 (56.7)	0.035
Peripheral vascular disease	111	7 (6.3)	4 (4.9)	3 (10.0)	0.330
Cerebrovascular disease	111	3 (2.7)	2 (2.5)	1 (3.3)	0.803
Connective tissue disease	111	13 (11.7)	12 (14.8)	1 (3.3)	0.095
Diabetes mellitus	111	13 (11.9)	7 (8.9)	6 (20.0)	0.109
Charlson index, median (IQR)	111	2 (1–4)	2 (1–3)	3 (2–4)	0.006
Donor information					
Age at the time of donation median (IQR)	111	48 (31–55)	48 (31–56)	48 (34–55)	0.750
Donation after circulatory death (DCD)	111	3 (2.7)	1 (1.2)	2 (6.7)	0.117
Donation after brain death (DBD)	111	108 (96.4)	79 (97.5)	28 (93.3)	0.292
Infectious donor risk					0.423
Standard	103	81 (78.6)	62 (80.5)	19 (73.1)	
Non standard	103	22 (21.4)	15 (19.5)	7 (26.9)	
Cause of donor death					0.362
Trauma	111	34 (30.6)	26 (32.1)	8 (26.7)	
Cerebrovascular	111	69 (62.2)	49 (60.5)	20 (66.7)	
Other	111	8 (7.2)	6 (7.4)	2 (6.7)	
Transplant information					
Days from inclusion list to transplant, median (IQR)	111	235 (83–508)	237 (83–534)	154 (76–349)	0.307
Single-lung	111	16 (14.4)	9 (11.1)	7 (23.3)	0.103
Double-lung	111	95 (85.6)	72 (88.9)	23 (76.7)	0.103
Heart + Lung	111	4 (3.7)	3 (3.8)	1 (3.4)	0.941
Ischemia Time (hours) median (IQR)	85	5.4 (4.35–6.1)	5.1 (4.3–6.4)	5.8 (4.8–6.1)	0.188
Primary graft non function	111	15 (14.0)	12 (15.2)	3 (10.7)	0.558
Delayed graft function	111	1 (0.9)	1 (1.3)	0 (0.0)	0.550
Induction regimen					
Bolus of steroids	111	104 (93.7)	75 (92.6)	29 (96.7)	0.433
Antylimphocyte globulin	111	11 (9.9)	7 (8.6)	4 (13.3)	0.463
Basiliximab	111	56 (50.5)	41 (50.6)	15 (50.0)	0.954
Maintenance regimen					
Cyclosporine	111	11 (9.9)	6 (7.4)	5 (16.7)	0.147
Azathioprine	111	13 (11.7)	9 (11.1)	4 (13.3)	0.746
Tacrolimus	111	73 (65.8)	59 (72.8)	14 (46.7)	0.010
Mycophenolate	111	37 (33.3)	29 (35.8)	8 (26.7)	0.365
Everolimus	111	1 (0.9)	1 (1.2)	0 (0.0)	0.541
Steroids	111	84 (75.7)	64 (79.0)	20 (66.7)	0.178
Etanercept	111	28 (25.2)	23 (28.4)	5 (16.7)	0.206
Positive Donor Derived Samples	111	59 (53.2)			
BSI	*59*	*3 (10.1)*	*1 (1.2)*	*2 (6.7)*	0.117
BAL	*59*	*56 (94.9)*	*41 (50.6)*	*15 (50.0)*	0.954
Recipient colonization	111	17 (77.3)			
CPE Rectal colonization	*16*	1 (0.9)	0 (0.0)	1 (3.3)	0.099
BAL	*16*	16 (14.4)	12 (14.8)	4 (13.3)	0.844
Perioperative Antibiotic prophylaxis					
Mono - regimen	111	26 (23.4)	21 (25.9)	5 (16.6)	0.306
*Piperacillin/tazobactam*	111	21 (80.7)	17 (80.9)	4 (80)	0.181
*Levofloxacin*	111	2 (7.7)	1 (4.7)	1 (20)	0.250
*Meropenem*	111	2 (7.7)	2 (9.5)	(0.0)	0.473
*Cefepime*	111	1 (3.8)	1 (4.7)	0 (0.0)	0.619
Combo - regimen	111	85 (76.6)	60 (74.1)	25 (8.3)	0.306
*Vancomicin* *-Cefepime*	111	5 (5.9)	2 (3.3)	3 (12.0)	0.099
*Piperacillina* */tazobactam_levofloxacin*	111	79 (92.9)	58 (96.6)	21 (84.0)	0.099
*Meropenem_levofloxacin*	111	1 (1.2)	0 (0.0)	1 (4)	0.099
Total Duration of PAP (median, IQR)	110	10 (6–13)	10 (6–14)	9 (6–10)	0.138
Duration of PAP ≤6 days	110	29 (27.1)	20 (25.6)	9 (31.0)	0.577
Recipient colonization after lung transplant	110	25 (22.7)	13 (16.2)	12 (40.0)	0.008
BAL	*25*	20 (18.0)	9 (11.1)	11 (36.7)	0.002
Rectal (CPE)	*25*	4(3.6)	3 (3.7)	1 (3.3)	0.926
Urinary	*25*	4 (3.6)	3 (3.7)	1 (3.3)	0.504
Time of colonization from Tx	*25*	15 (6–41)	17 (6–42)	10 (4–36)	0.695
Outcome					
ICU Readmission	111	15 (13.5)	6 (7.4)	9 (30.0)	0.002
ICU LOS	109	15 (6–26)	11 (6–24)	21 (15–33)	0.001
Duration of MV	110	6 (3–12)	4 (2–9)	9 (7–17)	<0.001
Renal Replacement Therapy	111	41 (36.9)	22 (27.2)	19 (63.3)	<0.001
Continous Renal Replacement Therapy	111	38 (34.2)	21 (25.9)	17 (56.7)	0.002
Days of Continous Renal Replacement Therapy median (IQR)	*36*	12 (8–21)	*10 (5–20)*	16 (11–21)	0.002
Re-IOT	106	17 (15.3)	10 (12.8)	7 (25.0)	0.132
Re-hospitalization	94	5 (4.5)	3 (4.5)	2 (7.4)	0.567
Death 30 days	111	18 (16.2)	14 (17.3)	4 (13.3)	0.616

Abbreviations: BAL, bronchoalveolar lavage; BSI, bloodstream infection; COPD, chronic obstructive pulmonary disease; CPE, carabapenem producing enterobacterales; DBD, donation after brain death; DCD, donation after circulatory death; ICU, intensive care unit; IQR, interquartile range; LOS, length of stay; MV, mechanical ventilation; OI, orotracheal intubation; PAP, Perioperative antibiotic prophylaxis.

Recipient BAL colonization was found in 16 (14.4%) patients, data shown in Supplementary Table S1. Donor characteristics are summarized in Table 1: median age at the time of donation was 48 years (IQR: 31–55). Mainly were donation after brain death (DBD) (107, 96.4%). Infectious risk was considered as “*non-standard*” in 22 (21.4%) donations and ≥1 positive sample from BAL and blood was obtained from 59 (53.2%) donors. Data about donor/recipient BAL colonization are shown in Supplementary Table S1.

**FIGURE 2:**
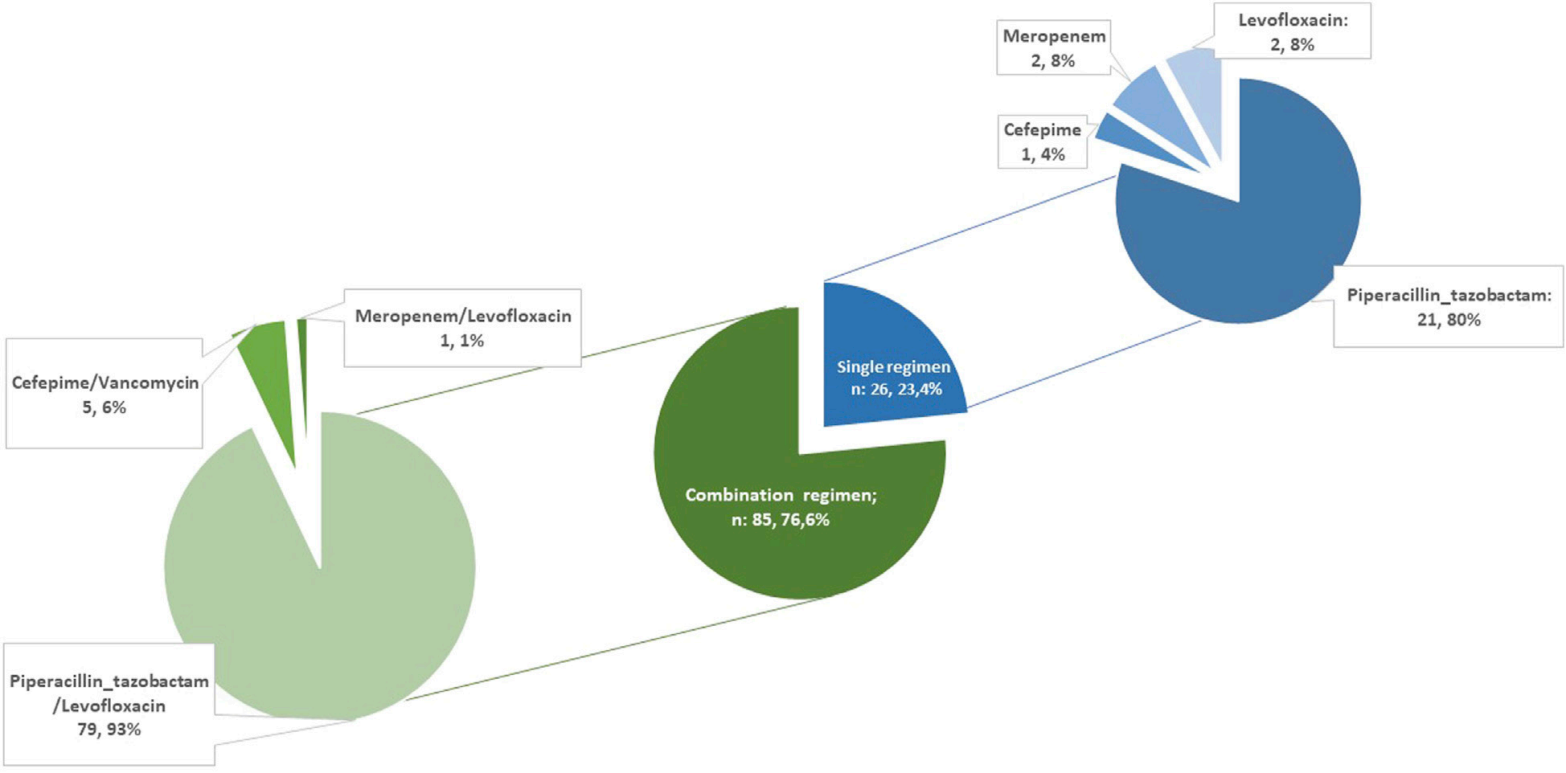
Perioperative antibiotic prophylaxis regimens.

PAP consisted of single-agent or combination regimens in 26 (25.2%) and 85 (74.8%) LuTR, respectively (Figure 2).

Piperacillin/tazobactam was the most common agent used either as monotherapy (n = 21, 80.7%) as combination with levofloxacin (n = 79, 92.9%). Among all, 11 patients did not receive piperacillin/tazobactam as backbone of peri-operative antibiotic prophylaxis (PAP), 8 due to drug allergy/intolerance (of which 2, 1 and 5 of them received meropenem, levofloxacin alone and cefepime, respectively) and 3 due to surgeon decision. Levofloxacin was not administered in 8 patients (in 3 cases due to reported allergy to fluoroquinolones and in 5 patients due to other contraindications - history of epilepsy n = 2, QT prolongation n = 2, connective tissue disease n = 1). Vancomycin was administered as part of the PAP regimen in two recipients due to MRSA colonization of the respiratory samples in the pre-transplant period. The median duration of antibiotic prophylaxis was 10 days (IQR 6–13). No differences were found in PAP duration according to donor sample and recipient colonization (Supplementary Table S2).

MDRO colonization after LuT is reported in Supplementary Table S3. No differences were found regarding MDRO colonization in patients with PAP ≤6 days or >6 days (9, 32.1% vs. 15, 19.2%, p = 0.161). No *Clostridioides difficile* infection was found in the entire patient cohort.

EPOIs were diagnosed in 30 (27%) patients: 22 (73.3%) pneumonia, 1 (3.3%) bloodstream infections (BSI) and 2 (6.6%) surgical site infections. The median time to EPOIs was 10 days (IQR 6–23) from LuT. Overall, 13 patients with EPOIs were still on PAP at the time of infection diagnosis and therefore the antibiotic treatment was changed and targeted based on antimicrobial susceptibility of the pathogens. Enterobacterales were the main pathogens, none had a DTR profile. Two EPOIs were considered as donor derived events, data shown in Supplementary Table S4. Trend of LuT performed and related EPOIs devolopment during the study period is showed in Supplementary Figure S1. Comparison of patients with and without EPOIs is shown in Table 1. Patients with EPOIs were more frequently male (22, 73.3% vs. 42, 51.9%, p = 0.042) with older age (54, IQR: 46–63 vs. 48, IQR: 36–59, p = 0.029) with more frequently IPF (14, 46.7% vs. 20, 24.7%, p = 0.026) as underlying lung disease. No differences were found as regard single or combination PAP regimens administered. There were no differences in 30-day mortality (4, 13.3% vs. 14, 17.3%, p 0.616). However, patients with EPOIs had more longer length of stay (LOS) in ICU (15 days, IQR:6–26 vs. 11 days, IQR: 6–24, p:0.001) and ICU readmission rates (9, 30% vs. 6, 7.4%, p = 0.002), longer duration of mechanical ventilation (9 days, IQR: 7–17 vs. 4 days, IQR: 2–9, p < 0.001) and more frequently need of renal replacement therapy (19, 63.3% vs. 22, 27.2%, p < 0.001).

The multivariable analysis of risk factors for EPOIs is showed in Table 2, panel A. No advantages were found for combination regimens compared to single-agent PAP in preventing EPOI (OR: 1.57, 95% CI: 0.488–5.068, p:0.448). The model was adjusted for male gender, Charlson Comorbidity Index, Tacrolimus as maintenance immunosuppresive regimen, idiopatic pulmonary fibrosis as leading cause for lung transplant and primary graft non function. To investigate the impact of PAP duration of EPOIs development, we excluded from analysis patients with ongoing antibiotic prophylaxis at infection diagnosis (13 of 30, 43%). The remaining 17 patients developing EPOIs were included in the model. PAP duration ≤6 days was used as main exposure variable. We didn’t find a significantly impact of PAP duration (OR:2.165, 95% CI: 0.596–7.863, p: 0.240) (Table 2, panel B). The multivariable analysis of risk factors for EPOIs was repeated by selecting only patients treated with piperacillin/tazobactam in monotherapy and in association with levofloxacin confirming no advantages for combination regimen compared to single-agent PAP in preventing EPOI and neither significantly impact of PAP duration (Supplementary Table S5, panel A and B).

**TABLE 2:** Multivariable binary logistic regression of: total EPOIs development at 30 days after lung transplantation (*Panel a*); EPOIs in patients without PAP at the time of infection diagnosis (*Panel b*).

Panel a	OR	IC 95%	P
Male gender	0.736	0.241–2.244	0.590
Idiopatic pulmonary fibrosis as leading cause for lung transplant	1.436	0.517–3.984	0.487
Primary graft non function	0.304	0.062–1.488	0.142
Charlson comorbidity index	1.236	0.916–1.667	0.167
Tacrolimus as mantainance regimen	0.295	0.095–0.918	0.035
PAP combination regimens	1.573	0.488–5.068	0.448
**Panel b**	**OR**	**IC 95%**	**P**
Male gender	0.854	0.194–3.756	0.834
Idiopatic pulmonary fibrosis as leading cause for lung transplant	0.466	0.111–1.955	0.297
Primary graft non function	0.149	0.013–1.671	0.123
Charlson comorbidity index	1.391	0.937–2.064	0.102
Tacrolimus as mantainance regimen	0.324	0.076–1.376	0.127
PAP combination regimens	5.606	0.643–48.901	0.119
Duration of PAP ≤6 days	2.165	0.596–7.863	0.240

Abbreviations: OR, odds ratio; IC, confidence intervals; PAP, perioperative antibiotic prophylaxis.

## Discussion

We analysed a large cohort of LuTRs to evaluate different PAP regimens used to prevent EPOIs, mainly with piperacillin/tazobactam as backbone. No differences were found as regard EPOIs development between combination or single agent PAP regimens. In addition, we observed a prolonged PAP not justified by donor/recipient culture results underlying the need of *ad hoc* strategies to limit the use of broad spectrum and unnecessary prolonged regimens.

The knowledge of the patient’s infectious risk is crucial for an appropriate management of LuTRs in the perioperative period. It may be helpful to consider targeted PAP for patients who are colonized with MDROs and, conversely, to limit the use of high microbiological impact antibiotics (i.e., carbapenems) if alternatives available. Regarding this aspect, characteristics of patients in our cohort are peculiar. The most frequent lung diseases requiring transplantation, COPD/emphysema and cystic fibrosis, are poor represented in our study [21]. Patients with COPD and cystic fibrosis suffer frequently of bacterial infections with consequently prolonged broad-spectrum antibiotics exposition and higher risk of MDROs colonization [3, 9]. Conversely, patients with interstitial lung disease show low rates of bacterial complication with a reduced antibiotic exposure and MDROs colonization [22]. Indeed, in our cohort, the rate of MDROs recipient colonization and infection was very low and PAP regimen was almost always effective on antimicrobial susceptibility profiles of donor/recipient isolates. We noted that, among all interstitial lung diseases collected in our center, patients with IPF appeared to have the highest risk of developing infections. Further studies are needed to confirm this finding.

The choice of single or combination PAP regimens is left to referral clinicians. If drugs with activity against MDR Gram negative rods are almost universally used, a second antibiotic with activity against *Staphylococcus aureus* could be added, according with local epidemiological data. In a large survey on perioperative antibiotic therapy in LuT involving 99 centers worldwide, most of the participants reported PAP regimens covering Gram negative rods with activity against *Pseudomonas aeruginosa. O*nly one-third of the centres targeted *S. aureus*, almost exclusively from the USA and against methicillin resistant *S.aureus* (MRSA) [7], with vancomycin as preferred drug. The low *S. aureus* rate and the absence of a clear benefit from using a combination regimen in our cohort, support the need to set PAP according with local epidemiology.

Finally, duration of PAP is another matter of debate. Ideally, PAP should be stopped as soon as cultures of the donor and the recipients are reported as negative to reduce the risk of MDROs selection and/or *C. difficile* infections [23]. However, it has been shown that PAP duration among transplant centers is very heterogeneous [7]. In our study, PAP duration was unacceptably too prolonged even in cases without donor/recipient positive cultures, deviating from internal guidelines. Although with few cases, our analysis shows that a prolonged PAP is not protective for EPOIs development, thus supporting the opportunity of shortening PAP duration. In this regard, it seems desirable to design and/or standardize *ad hoc* antimicrobial stewardship strategies to avoid unnecessary prolonged PAP in lung transplant recipients in our center [23, 24].

There are several limitations in this study. First, we collected data from a single-center cohort of LuTR over a 20-year study period, however PAP regimens and approach to EPOI diagnosis did not change across years. Furthermore, our patients suffered mainly from interstitial lung disease and cystic fibrosis was not represented. Both these limitations could limit the generalizability of our results. However, our findings could add evidence supporting prophylaxis with a single drug in LuTRs with non-cystic fibrosis/COPD as underlying disease. Moreover, the rate of donor derived events could have been be underestimated due to the lack of respiratory sample in around half of the donors. In addition, the limited sample size and the heterogeneity of PAP administration did not allow to infer any conclusion about the impact of prophylaxis duration on EPOIs development. Finally, the retrospective design of the study could have reduced the accuracy of data collection. However, we attempted to reduce this limitation by thorough data quality control.

Despite these limitations, our results suggest no advantages for combination regimens over a single-agent regimen in preventing EPOIs in LuTRs with interstitial lung diseases as underlying disease. However, further studies are needed to confirm this hypothesis.

## Data Availability

The raw data supporting the conclusions of this article will be made available by the authors, without undue reservation.
