# Supplementary figures and images for: Antibiotic Prophylaxis in Patients Undergoing Lung Transplant: Single-Center Cohort Study
Source: Transpl Int. 2024 Aug 16;37:13245. doi: 10.3389/ti.2024.13245 (PMC11361928; doi:10.3389/ti.2024.13245)

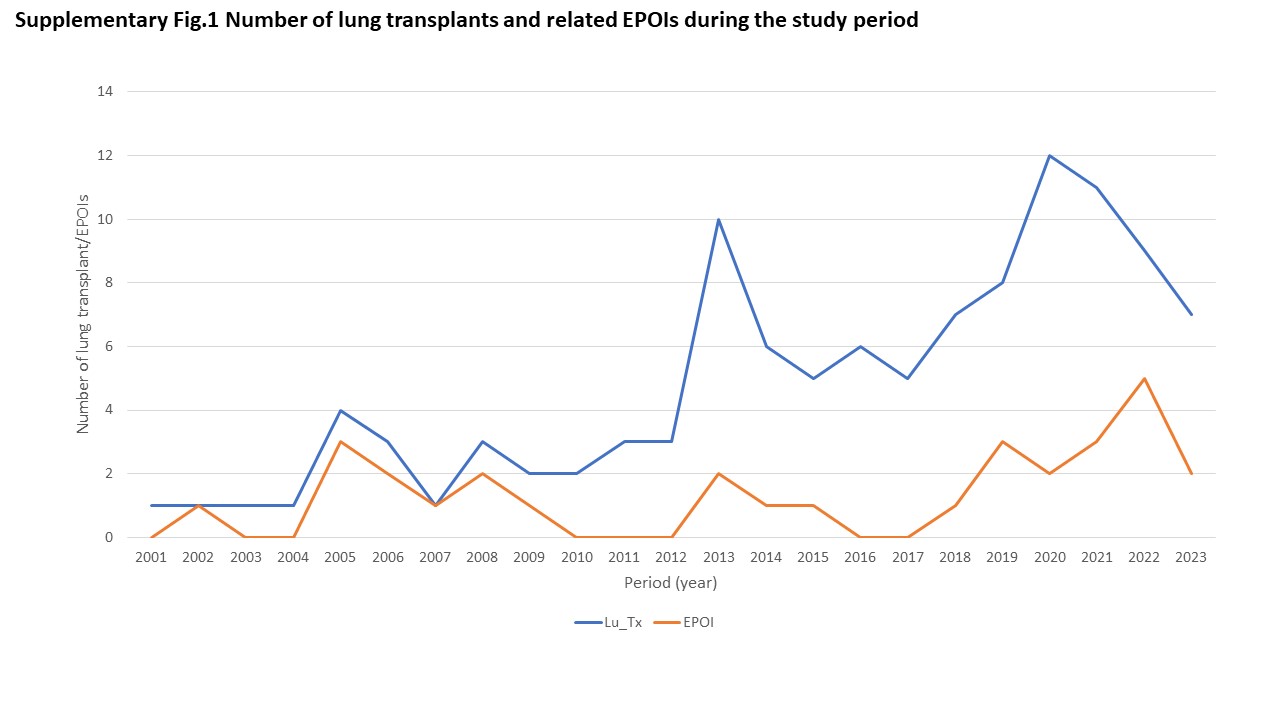

Supplement: Supplementary file 1 [file Image1.JPEG]
