# Supplementary material for: Antibiotic Prophylaxis in Patients Undergoing Lung Transplant: Single-Center Cohort Study
Source: Transpl Int. 2024 Aug 16;37:13245. doi: 10.3389/ti.2024.13245 (PMC11361928; doi:10.3389/ti.2024.13245)
Supplement: Supplementary file 2 [file DataSheet1.docx]

**Supplementary Tables**

**Supplementary table 1** Pre-transplant Recipient and Donor BAL Colonization

|  | Donor BAL Colonization: 56 (%) | Pre-transplant Recipient BAL Colonization: 16 (%) |
| --- | --- | --- |
| **Gram negative** |  |  |
| *Klebsiella spp* | 9 (16.1) | 2 (12.5) (*1: 3GC-R*) |
| *Escherichia coli* | 3 (5.3) | 1 (6.25) |
| *Enterobacter spp* | 3 (5.3) | 0 (0.0) |
| *Citrobacter spp* | 4 (7.1) | 0 (0.0) |
| *Serratia spp* | 3 (5.3) | 0 (0.0) |
| *Acinetobacter spp* | 2 (3.5) | 0 (0.0) |
| *Pseudomonas spp* | 5 (8.9) | 1 (6.25) |
| *Stenotrophomonas spp* | 2 (3.5) | 0 (0.0) |
| *Haemophylus spp* | 10 (17.8) | 1 (6.25) |
| **Gram positive** |  |  |
| *Staphylococcus aureus* | 27 (48.2) | 8 (50.0) |
| *MRSA* | 2 (3.5) | 2 (12.5) |
| *Streptococcus pneumoniae* | 2 (3.5) | 0 (0.0) |
| Other isolates † | 3 (8.9) | 1 (6.25) |

Abbreviations: 3GC-R: Third-generation cephalosporin resistance ;BAL: Bronchoalveolar lavage; CPE: Carbapenem producing *Enterobacterales*; MRSA: Methicillin resistant *Staphylococcus aureus*.

† Other BAL isolates for Donor include: 2 *Hafnia alvei*, 1 *Veillonella spp*.*;* Other BAL isolates for Recipient include: 1 Geotrichum spp*;*

**Supplementary Tab 2.** Duration of antibiotic prophylaxis according to donor sample and pre-transplant recipient colonization

|  | **< 6 days** | **6-9 days** | **>9-13 days** | **>13days** |
| --- | --- | --- | --- | --- |
| **Donor Sample positive** † **(59/111)** | 13/59 (22.0) | 14/59 (23.7) | 17/59 (28.8) | 14/59 (23.7) |
| **Recipient Colonized (17/111)** | 4/17 (23.5) | 4/17 (23.5) | 6/17 (35.2) | 3/17 (17.6) |

† Duration of antibiotic prophylaxis for one patient with Donor sample positive was not availabe

**Supplementary Table 3.** MDRO colonization of patients after lung transplantation

|  | **Urine colonization**  **(4/25)** | **BAL colonization**  **(20/25)** | **Rectal colonization**  **(4/25)** |
| --- | --- | --- | --- |
| **Enterobacterales** |  |  |  |
| ***Enterobacter spp*** |  | **1** |  |
| *3GC-R* |  | *1* |  |
| ***E.coli (3GC-R)*** | **2** | **3** |  |
| *FQ-R* | *1* |  |  |
| *3GC-R* | *1* | *3* |  |
| ***K.pneumoniae*** | **1** | **10** | **4** |
| *3GC-R* | *1* | *9* |  |
| *CR (KPC producing)* |  | *1* | *4* |
| **Non fermentative Gram negative** |  |  |  |
| ***Pseudomonas spp*** |  | **4** |  |
| *Wild Type* |  | *1* |  |
| *Cef-R* |  | *2* |  |
| *CR* |  | *1* |  |
| **Other** † |  | 3 |  |

Abbreviations: 3GC-R: Third-generation cephalosporin resistance; Cef-R: cephalosporin resistance; CR: carbapenem-resistance; FQ-R: Fluoroquinolone-resistance; MDRO: multi-drug resistant organisms.

† Other include: 2 Achromobacter xylosoxidans *3GC-R; 1 Stenotrophomonas maltophilia.*

**Supplementary Table 4.** Early post-operative infections at 30 days after lung transplantation

|  | **Surgical Site Infection**  **(2/30)** | **BSI**  **(1/30)** | **BSI+Pneumonia (4/30)** | **Pneumonia**  **(22/30)** | **Total** |
| --- | --- | --- | --- | --- | --- |
| **GRAM NEG** |  |  |  |  |  |
| **Enterobacterales** |  |  |  |  |  |
| ***E.coli (3GC-R)*** | **1** |  | **1** | **2** | **4** |
| ***K.pneumoniae*** |  | **1** | **1** | **3** | **5** |
| *Wild Type* |  |  |  | *2* |  |
| *3GC-R* |  | *1* |  | *2* |  |
| *CR* |  |  | *1* |  |  |
| **Non fermentative Gram negative** |  |  |  |  |  |
| ***Pseudomonas spp*** |  |  | **2** | **3** | **5** |
| *Wild Type* |  |  |  | *1* |  |
| *Cef-R* |  |  | *2* | *2* |  |
| *FQ-R* |  |  | *1* | *1* |  |
| **GRAM POS** |  |  |  |  |  |
| **MRSE** | **1** |  |  |  | **1** |
| ***E.faecium*** | **1** |  |  |  | **1** |
| **Other** † |  |  |  | **1** | **1** |
| **No isolates** |  |  |  | **13** | **13** |

Abbreviations: 3GC-R: Third-generation cephalosporin resistance; BSI: bloodstream infection; Cef-R: cephalosporin resistance; CR: carbapenem-resistance; FQ-R: Fluoroquinolone-resistance; MRSE methicillin-resistant *Staphylococcus epidermidis*;† Other include: Donor Derived Tuberculosis

**Supplementary Tab.5** Multivariable binary logistic regression in patients receiving piperacillin/tazobactam as monotherapy and as combination with levofloxacin of : total EPOIs development at 30 days after lung transplantation (*Panel a*) ; EPOIs in patients without PAP at the time of infection diagnosis (*Panel b*).

|  | | | |
| --- | --- | --- | --- |
| **Panel a** | **OR** | **IC 95%** | **P** |
| Male gender | 0.926 | 0.279-3.071 | 0.900 |
| Idiopatic pulmonary fibrosis as leading cause for lung transplant | 1.220 | 0.395-3.770 | 0.730 |
| Primary graft non function | 0.307 | 0.057-1.656 | 0.170 |
| Charlson comorbidity index | 1.354 | 0.971-1.888 | 0.074 |
| Tacrolimus as mantainance regimen | 0.217 | 0.061-0.768 | 0.018 |
| PAP combination regimens | 2.045 | 0.479-8.736 | 0.334 |
|  | | | |
| **Panel b** | **OR** | **IC 95%** | **P** |
| Male gender | 0.898 | 0.270-2.986 | 0.860 |
| Idiopatic pulmonary fibrosis as leading cause for lung transplant | 1.156 | 0.371-3.608 | 0.802 |
| Primary graft non function | 0.361 | 0.066-1.970 | 0.239 |
| Charlson comorbidity index | 1.366 | 0.978-1.907 | 0.067 |
| Tacrolimus as mantainance regimen | 0.202 | 0.054-0.762 | 0.018 |
| PAP combination regimens | 2.069 | 0.481-8.900 | 0.329 |
| Duration of PAP ≤ 6 days | 0.840 | 0.251-2.813 | 0.777 |

Abbreviations: OR, odds ratio; IC: confidence intervals; PAP: Perioperative Antibiotic prophylaxis
